# The Use of Radio and Telemedicine by TMAS Centers in Provision of Medical Care to Seafarers: A Systematic Review

**DOI:** 10.3390/jpm13071171

**Published:** 2023-07-22

**Authors:** Gopi Battineni, Nalini Chintalapudi, Giulio Gagliardi, Francesco Amenta

**Affiliations:** 1 Clinical Research Centre, School of Medicinal and Health Products Sciences, University of Camerino, 62032 Camerino, Italy; nalini.chintalapudi@unicam.it (N.C.);; 2 Research Department, International Radio Medical Centre (C.I.R.M.), 01444 Rome, Italy

**Keywords:** seafarers, TMAS, medical devices, maritime telemedicine, satellite technology, medical assistance at sea

## Abstract

*Objective*: From medicine via radio to telemedicine, personalized medical care at sea has improved significantly over the years. Currently, very little research has been conducted on telemedicine services and tools at sea. This study aims to review real-time case studies of seafarers’ personalized treatment via telemedical devices published in medical journals. *Methods*: A literature search was conducted using three libraries such as PubMed (Medline), Cumulative Index to Nursing and Allied Health Literature (CINAHL), BioMed Central, and Google Scholar. The Medical Subject Headings (MeSH) were used for information retrieval and document selection was conducted based on the guidelines of preferred reporting items for systematic reviews and meta-analyses (PRISMA) 2020 flowchart. Selected articles were subjected to quality checks using the Newcastle–Ottawa scale (NOS). *Results*: The literature search produced 785 papers and documents. The selection was conducted in three stages such as selection, screening, and inclusion. After applying predefined inclusion and exclusion criteria, only three articles on real-time medical assistance with telemedical tools were identified. It is reported that medical attention is delivered to seafarers in real time thanks to advancements in telemedicine, satellite technology, and video conferencing. *Conclusions*: By improving the quality of medical care and reducing response times for medical emergencies at sea, lives have been saved. There are still several gaps despite these advancements. Medical assistance at sea should therefore be improved to address many of the still unsolved issues.

## 1. Introduction

The history of medical assistance at sea dates back to the early days of ocean exploration when mariners encountered various health hazards and medical emergencies with no possibility of being properly treated due to the lack of onboard health professionals. The development of radiotelegraphy by Guglielmo Marconi in 1897 has allowed us to provide medical assistance to ships from ashore medical centers or ships with medical facilities on board [1]. The realm of medical assistance at sea has evolved over the past century, largely owing to advancements in radio communication and, subsequently, telemedicine [2]. However, the medical assistance available to seafarers is often basic and limited to the use of elementary medical equipment, and non-advanced communication systems, such as, in the past, radio telegrams, and, currently, conventional e-mail or telephone calls.

With the development of radiotelegraphy and later radio telephony, medical professionals were able to transmit instructions and guidance to seafarers experiencing medical emergencies [3,4]. The use of internet-based communication systems, such as video conferencing, has enabled remote medical treatment to be provided to patients at sea more effectively and efficiently than ever before. These advancements in telecommunications have ensured swift and effective medical assistance along with the ability of providing medical services in the maritime environment. This approach has remarkably changed the provision of medical assistance at sea over time and has become relevant to ensure the safety and well-being of those on board.

Delivering medical assistance at sea poses several challenges and issues due to the unique environment and logistical constraints. Ships, especially those on long voyages or in remote areas, can be far from medical facilities or specialized care. This makes it difficult to access timely medical assistance in case of need. Having limited medical resources and specialized equipment can hinder the delivery of comprehensive medical care onboard. Also, limited or unreliable satellite connections can hinder real-time consultations with onshore medical professionals and delay diagnosis and treatment. In case of medical emergencies, timely evacuation to a shore-based medical facility may be necessary. However, arranging medical evacuations at sea can be logistically complex and expensive, especially in adverse weather conditions. 

The use of telehealth on onboard ships helps deliver medical help minimizing the challenges and burdens encountered by seafarers. This is due to the limited medical knowledge of ship masters or onboard medical staff. Telehealth can also improve monitoring, timeliness, and communication within healthcare systems. Telemedical Maritime Assistance Service (TMAS) centers provide medical services based on radio codes through different channels such as email, marine radio, telephone, video conferences, and fax [5,6,7]. TMAS centers and seafarers began to use telehealth more frequently, especially, during the recent pandemic to reduce in-person contact [8,9]. The doctors at TMAS centers communicate over the phone, email, or video, which is beneficial for both patient health and practice [10,11]. Using digital medical devices, vitals are gathered, progress is monitored, external lesions can be viewed, and images of skin, ears, eyes, etc., can be captured and evaluated. The availability of digital devices like these has increased the effectiveness of telemedicine, overcoming the handicap of inaccurate information provided from the ship’s side. 

In the past few decades, telemedicine has further transformed the field, enabling the remote medical diagnosis and treatment of patients in maritime environments. A literature review of telemedical assistance in the marine industry domain has never explored the previous objectives in detail. Therefore, we will be able to provide a broader understanding of the area and facilitate future technological applications. Only two reports gave an overview of patient safety and legal impacts [12,13]. Another study gave an overview of European remote medical care services at sea from a variety of communication channels and generational perspectives [14]. 

As far as we know, no comprehensive study has focused on contextualizing data collection and medical operations between ships and TMAS centers using radio communications and telemedical devices. The purpose of this study was to explore the evolution of medical assistance at sea, from primitive radio-based methods to modern telemedicine. A discussion of existing medical assistance case studies has also been provided, along with the most important aspects, including techniques and communication tools.

## 2. Methodology

### 2.1. Document Search

The document search was conducted via different medical databases, such as PubMed (Medline), Cumulative Index to Nursing and Allied Health Literature (CINAHL), BioMed Central (BMC), and Google Scholar. The Preferred Reporting Items for Systematic Reviews and Meta-Analysis (PRISMA) 2020 guidelines were followed for document selection to promote transparency, completeness, and clarity in research reporting [15]. Medical Subject Headings (MeSH) terms are used for document indexing and retrieval. The search keywords ‘telemedicine’, ‘seafarers’, ‘maritime industry’, ‘marine telehealth’, ‘seafarers health’, ‘radio medicine’, ‘remote telemonitoring’, ‘maritime telemedicine’, and ‘medical assistance at sea’ were applied to identify real-time case studies where medical assistance at sea was carried out. Boolean operators like ‘AND’ and ‘OR’ were included in search databases to acquire variations in the vocabulary and for a better search strategy. Table 1 presents the document count retrieved from different databases. 

### 2.2. Inclusion Exclusion Criteria

Only documents published in the English language were considered. All the identified articles included a case study in which medical assistance at sea with tools or techniques was applied. The degree of analysis was not a significant inclusion criterion. Articles that did not include evidence of a case study with a clear motivation to protect seafarers from a medical point of view were included. Review articles and books were excluded from the study. Case studies on seafarers’ blood sample data, onsite doctor visits, autopsy reports, and clinical pathways were excluded. Non-peer-reviewed, unpublished, conference proceedings, abstracts, and publications in languages other than English were also not considered.

### 2.3. Quality and Risk Bias Assessment

During the review process, each author independently screened papers and assessed their quality and relevance to the research. Papers were selected based on consensus among reviewers and conformity to inclusion and exclusion criteria. Upon reading the abstracts, the authors compiled a list of articles that they believed qualified in primer appraisal. Study quality was assessed using the Newcastle–Ottawa scale (NOS), which is a quality assessment tool that ranks studies and assesses the risk of bias [16]. It is fairly simple to interpret the NOS scale, which rates studies as poor (0–4), fair (5–6), and good (7–9). The studies that scored NOS ≥ 7 were considered for final review. 

## 3. Results

The review findings and study characteristics, including interventions and outcomes, are presented in this section. We also summarize the key findings derived from the research across the studies in terms of providing medical assistance at sea.

### 3.1. Document Selection Flowchart

**Figure 1:**
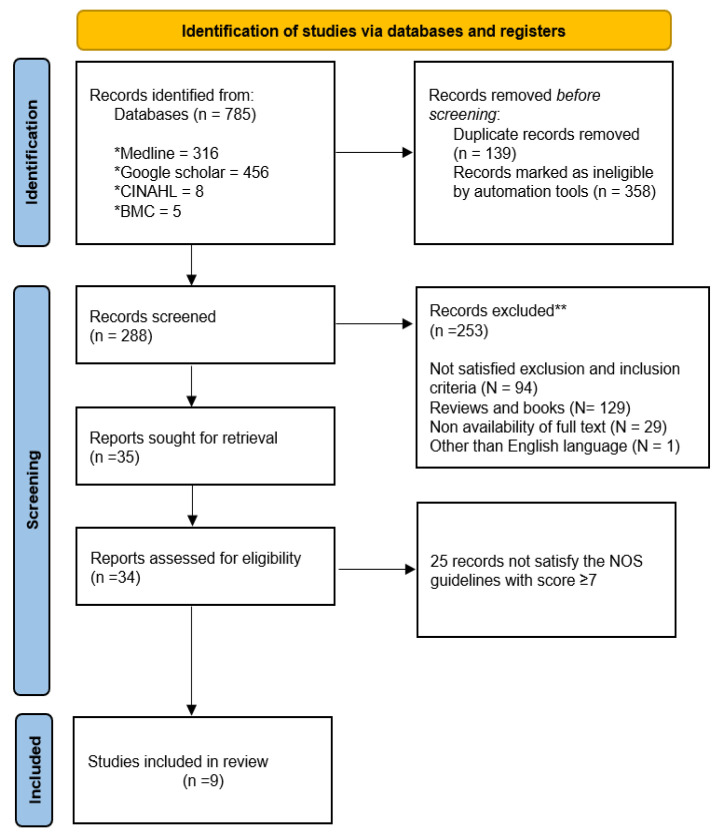
PRISMA 2020 flow diagram for new systematic reviews which included searches of databases only (* searched database).

Figure 1 provides a PRISMA flow diagram for selected 785 articles among the given searched libraries. A careful review of the title and summary of each study revealed that 358 papers were ineligible; 139 were withdrawn because there were duplicates, and three did not have enough data. In total, 288 studies were screened based on the inclusion and exclusion criteria. Of these, 253 articles were excluded because they did not satisfy the exclusion and inclusion criteria (n = 94), they were reviews and books (n = 129), due to the unavailability of full text (n = 29), and because they were written in a language other than English (n= 1). Ultimately, 35 papers met the eligibility criteria for the review. Before reaching the final selection, the previously mentioned search words were examined for differences, and when one word was different from others of a similar kind, various thoughts were discussed. By considering NOS criteria, the authors gathered information helpful to the exploration effort by reading all the articles before choosing those that were qualified to be used for the study. Ultimately, nine studies were included in the final review because other studies did not provide the motivation behind the case study presentation, news synopsis, and meta-analysis.

### 3.2. Communication Channels

**Figure 2:**
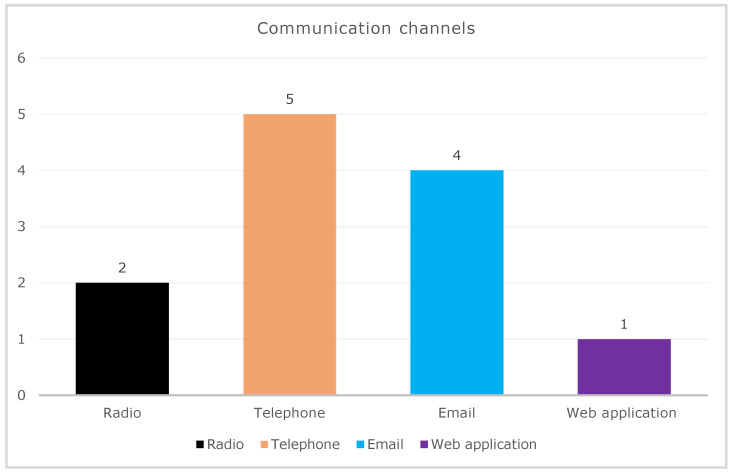
Different communication technologies are used for requests of medical assistance at sea.

By analyzing the studies that were finally selected, we identified that most seafarers contact TMAS centers for medical help through different means of communication. Figure 2 presents the frequency of work associated with distinct communication channels or telemedical technologies that were used by ships to contact onshore doctors. Five studies adopted the telephone [8,17,18,19,20], followed by email [8,18,19,21], radio [22,23], and web application platforms [24].

### 3.3. TMAS Centers and Means Communication

Maritime emergencies, illnesses, accidents, and other incidents that require medical advice, based on the captain’s decision, are managed by TMAS centers. Providing seafarers with health care is an essential part of their mission. TMAS centers in Europe conducted the majority of studies, while only one study was conducted in the US (Table 2). It appears that the Italian TMAS center, the International Radio Medical Center (C.I.R.M.), is one of the most populated in Europe. Additionally, seafarers contact these centers by telephone or by email to transfer their medical data, unlike RMD and CCMM, which use radios.

### 3.4. Study Characteristics

The major characteristics of examined studies based on study type, number of patients, year published, and observations are presented in Table 3. There was no evidence of studies reporting the telemedical assistance of seafarers with case studies before 2015. One study published in 1980 highlighted the transformation of medical messages from the casualty officer of the Royal Naval Hospital Plymouth via Portishead Radio [11]. This study analyzed the range of medicines prescribed for seafarers’ medical problems. Several diseases have been linked to diet-associated factors, such as alcohol and obesity. Because the full free text was not available, it was not included in the final survey. 

#### 3.4.1. Medical Assistance via Radio

Radio was the first telecommunication system used to provide medical assistance at sea. It became an essential tool for ship captains in the early 20th century, allowing them to inform onshore centers and authorities about medical issues aboard their ships. During the literature search, two published works were found on radio medical assistance for seafarers. One study from the Denmark TMAS center (i.e., RMD) adopted radio-telephone communication to analyze the injury patterns of workers and the factors that contribute to injuries [22]. The system allowed ship captains to communicate with a medical doctor onshore and receive immediate medical advice over the radio. This allowed physicians to guide ship captains, who were responsible for providing emergency medical care to injured crews and passengers. 

Five case studies presented by the French TMAS center (CCMM) illustrated the use of radiotelegraphy to diagnose and follow up on different medical conditions among seafarers living on ships [23]. To advance these practices, there is a need to develop video-conferencing technology. It is mentioned that radio calls enabled ships in the middle of the ocean to communicate effectively with medical personnel ashore, reducing the number of medical emergencies and deaths at sea. 

#### 3.4.2. Telemedicine Technologies

**Figure 3:**
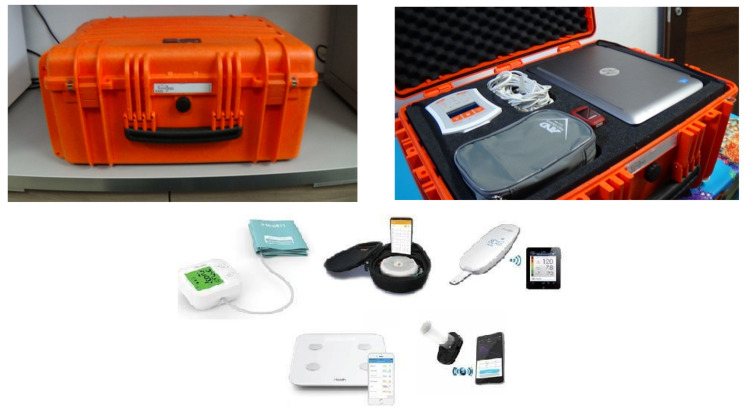
On-board telemedicine case containing medical devices courtesy of TelePharmaTec, the spin-off of Camerino University, Italy.

Seven studies are associated with the use of telemedicine, where medical professionals can now communicate with ships (via email, telephone, video conferencing, and web applications). This allows them to diagnose and treat medical conditions in real time [8,17,18,19,20,21,24]. Ships can contact TMAS by phone or by e-mail to provide a verbal description of a crew member’s medical condition, followed by an email with images. There are, however, still several problems to be solved. In general, the doctor ashore does not talk directly with the patient or does not see him, as contact is mediated through the captain or the ship’s officer with medical duties on board. This does not help to establish the fundamental patient-doctor relationship in the provision of medical assistance. On the other hand, the medical skills of the people with duties of medical assistance at sea are quite limited, and therefore this makes the delivery of medical assistance mediated by a third person complex. In an epidemiological study of seafarers’ injuries, telemedicine was found to provide remote access to medical care for those suffering from health issues while at sea [24]. With telemedicine, physicians can provide medical consultations and treatment to individuals aboard ships or boats via secure remote connections. For instance, with telemedical devices (presented in Figure 3), medical information (or knowledge) can be shared between sailors and onshore doctors. Some studies have shown that physicians can examine and diagnose medical problems miles away from patients via telephone communications from a TMAS center [17,20]. Handling seafarers’ medical records with email technology allows healthcare workers access to patient medical records, which helps determine appropriate treatment protocols [8,19,21]. In this way, seafarers obtain in-depth medical care regardless of where they are, and individuals at sea continue to benefit from telemedicine as technology advances.

Different contact possibilities are currently available for medical assistance at sea. The most popular is the store and forward method, which involves taking images or videos of a patient and transmitting them to a specialist for consultation [19,20,25]. Live telemedicine involves real-time audio-visual interaction between the provider and patient, similar to a video call. Remote patient monitoring involves collecting data from medical devices worn by patients and transmitting them to healthcare providers for analysis. This allows healthcare professionals to properly and continuously monitor patients’ health status and intervene, if necessary. Mobile health (mHealth) uses mobile devices such as smartphones and tablets to remotely deliver healthcare services [26,27]. These include educational resources, appointment scheduling, and virtual doctor visits. As these studies do not align with the research objectives, they were therefore excluded from the final survey. Telemedicine is becoming an increasingly valuable tool for medical assistance at sea as technology advances.

Adopted works in this review have highlighted the implementation of ship-to-shore video conferencing, which has revolutionized the provision of medical assistance at sea [23,24]. Through this technology, medical officers onboard can communicate with onshore doctors in real time, allowing a more accurate diagnosis and treatment. Medical personnel on board can consult with experienced professionals onshore through video streaming and reliable communication channels. In this way, ships without qualified health professionals can still obtain high-level medical care and transfer at-risk patients to ashore medical facilities. Ship-to-shore video conferencing has greatly improved the lives of mariners, ensuring timely and effective medical treatment and reducing the risks associated with remote treatment.

## 4. Discussion

Medical assistance at sea has come a long way since its inception with radio-based medicine. The procedures used over the last century at TMAS centers for seafarers’ healthcare are still in use today. Now onboard medical personnel can consult specialists onshore, share vital signs, and receive training via real-time audio and video communication. Our study explored the use of medical assistance via radio and telemedical devices during emergencies which have revolutionized the way patients at sea were treated. Medical professionals can guide crew members on board ships with limited medical equipment and resources [28,29].

Telemedicine has further enhanced the delivery of medical care at sea through real-time communication with onshore healthcare facilities [30]. Telemedical solutions provide remote access to healthcare professionals, electronic medical records, and consultations via satellite communications. Medical care at sea requires reliable medical care, which makes telemedicine increasingly important. The maritime industry relies on it to provide rapid medical assistance during emergencies, as well as ensure seafarers’ safety.

TMAS centers provide remote healthcare services that allow maritime patients to receive medical care and advice from medical professionals located on land in real-time [30]. It ensures that medical advice and diagnosis can be received even on vessels far away from medical facilities. Using telemedicine, medical professionals on land can visit patients using images, vital signs, and video conferences and consequently prescribe treatments. The improvement of medical assistance provided by telemedicine has also been shown to reduce medical evacuations, prolong major interventions, and save lives [31]. The use of telemedicine has revolutionized the delivery of medical care to those at sea.

The advent of satellite technology has significantly expanded the geographic availability of medical assistance, enabling healthcare providers to reach remote and isolated destinations. As maritime activities continue to increase in a globalized world, medical assistance at sea has become increasingly indispensable. With the ongoing advancements in technology and the expanding role of telemedicine, it is poised to play an even greater role in the protection of seafarers’ health in the future. With the continuous development of technology, healthcare providers can now rely on telemedicine to monitor patients’ conditions and manage medical emergencies in real-time. Efforts should be carried out to provide seagoing vessels with appropriate telemedicine equipment and to guarantee that solutions provided by different producers work on the maritime environment and that their friendly use would allow the proper transmission of biomedical data ashore.

### 4.1. What Needs to Be Done?

Telemedicine systems have advanced to include high-resolution video conferencing capabilities, which make it possible for diagnosis, treatment, and consultation to be conducted remotely [32,33]. By using these technologies, onshore medical practitioners can provide direction and guidance to onboard medical officers. Telemedicine ensures that crew members and passengers have access to qualified health care with accurate medical advice and support [34,35]. Additionally, it reduces unnecessary rescue operations and costs. Hence, telemedicine is an efficient, cost-effective, and beneficial method to deliver medical assistance to ships. 

#### 4.1.1. Integration of Artificial Intelligence (AI) in Telemedicine

The integration of artificial intelligence (AI) has provided telemedicine with the ability to offer a more personalized approach to diagnosis and treatment. The establishment of AI-based telemedicine systems for maritime vessels has numerous benefits. An AI-based marine doctor system enables prompt and efficient medical assistance to seafarers and crew members who encounter health complications [25]. Physicians can make informed decisions based on AI algorithms that analyze patient histories, laboratory results, and imaging studies [36]. Medical professionals will be able to make more accurate diagnoses, provide better treatment plans, and improve patient outcomes. It is important to address concerns regarding data privacy and ethical considerations regarding AI in telemedicine. AI has the potential to revolutionize telemedicine and enable healthcare providers to reach remote and underserved populations with high-quality medical care.

#### 4.1.2. Satellite Telecommunications

Medical assistance at sea has been remarkably improved by satellite communication. Communication channels can be established between vessels and land-based medical professionals, allowing for real-time consultations, diagnosis, and intervention on-site [37,38]. Satellite networks can transmit large amounts of data, including high-definition images and videos, which are essential for accurate diagnosis and medical support [39]. Satellite connections also allow remote monitoring of vital signs and other health parameters, making it possible for medical professionals to track the health status of patients in real-time.

Telemedicine consultations in case of medical issues on board are becoming increasingly popular for providing health care at sea. Using this approach, doctors can provide advice and guidance to seafarers from their locations using video conferencing. This method of healthcare delivery is especially helpful for remote vessels that lack onboard medical facilities and personnel [40]. In emergencies, telemedicine consultations can help diagnose and treat illnesses and injuries in real-time, reducing the need for medical evacuation. As technology continues to improve, telemedicine will become more accessible and affordable for mariners, providing them with much-needed medical assistance at sea. Despite these benefits, there are still challenges to overcome, such as connectivity issues and regulatory hurdles. There is also a need for greater diffusion of telemedical devices on ships and specific training of ship medical officers in telemedicine.

### 4.2. Implications of Medical Assistance at Sea in Healthcare

The implications of medical assistance at sea are numerous and far-reaching in the field of healthcare. The ability to provide medical care remotely, particularly in critical situations where time is essential has proven to be lifesaving in many instances [37,41,42,43]. The advancement of technology has enabled healthcare professionals to virtually reach patients in remote areas, improving diagnosis, treatment, and outcomes. The use of telemedicine can also reduce healthcare costs by avoiding expensive and potentially risky emergency medical evacuations. Additionally, telemedicine can improve access to health care in underserved areas. Healthcare providers and policymakers will need to address questions of efficacy and ethics as remote medical services expand.

### 4.3. Regional Context and Digital Divide Issues

Telemedicine is a rapidly growing field that has the potential to revolutionize healthcare delivery, especially in remote and underserved regions. It is important to consider the regional context and digital divide issues when considering telemedicine for sailing seafarers.

The availability and accessibility of telemedicine services can vary significantly across different regions. Seafarers may already be able to access telemedicine services in coastal areas or regions with a strong maritime industry, such as major port cities [44]. A dedicated telemedicine center or clinic should be properly equipped to provide remote consultations, diagnoses, and even certain treatments to seafarers at sea in such regions. On the other hand, in less developed or remote coastal regions, the availability of telemedicine services may be limited. Telemedicine may not be feasible in areas lacking reliable internet connectivity or advanced medical equipment [45]. Telemedicine for seafarers would require significant infrastructure development and capacity building.

The digital divide refers to the gap in access to and utilization of digital technologies between different populations or regions [46]. Seafarers who work on vessels operating in international waters or regions with limited connectivity may face difficulties in accessing telemedicine services due to unreliable or nonexistent internet connections [47]. The digital divide requires collaborative efforts from the government, maritime industry bodies, and telecommunications companies. By improving internet infrastructure and providing training programs for seafarers, telemedicine services can be made more accessible to all. Consequently, while telemedicine can provide valuable medical care to sailing seafarers, regional context and digital divide issues need to be considered. By addressing disparities in internet access and telemedicine infrastructure in a particular region, seafarers can access effective healthcare.

### 4.4. Limitations

This study typically focuses on specific research questions or interventions. As such, there is a chance it is not addressing all relevant aspects of the topic, potentially overlooking important outcomes, subgroups, or alternative interventions. Due to a limited number of studies the applicability and review findings with a special group of population, settings, or contexts may be limited. Because the included studies have focused on seafarers, it makes it challenging to generalize the findings to broader populations or different healthcare systems. Despite these limitations, we are confident that this study could contribute to the synthesis of evidence and provide an overview of the available literature.

## 5. Conclusions

In modern medical care at sea, telemedical assistance is an essential component, allowing for quick and accurate diagnosis, timely treatment, and improved patient outcomes. Although medical assistance at sea has been available for more than 100 years, little research has been carried out on its evaluation. In this study, we examined telemedicine’s role in maritime healthcare and examined how it has evolved. Throughout this review, we discussed technological advancements in medical assistance at sea. Since radio medical assistance was introduced in the past, telemedicine has improved the quality of healthcare for seafarers worldwide.

Despite its current limitations, telemedicine is set to continue improving medical assistance onboard ships in the future. More accurate remote diagnosis and treatment may be possible for doctors and other health professionals with advanced technology. Sophisticated monitoring systems and medical equipment can contribute to achieving this goal. As a result of AI, accurate predictions of illnesses and identification of high-risk patients could be achieved with significant benefits. Research and development investment by government agencies is crucial for improving healthcare service delivery.

## Figures and Tables

**Table 1:** Document search count among different databases.

*N*	Databases	Document Count
1.	PubMed (Medline)	316
2.	CINAHL	8
3.	BMC	5
4.	Google Scholar	456

**Table 2:** List of TMAS centers associated with the present study.

TMAS Centre	Location and Country	Means of Communication
Med solutions international	New York, USA	Email
RMN	Bergen, Norway	Telephone
CCMM	Toulouse, France	Radiotelegraphy, Telephone
UCMTM	Gdynia, Poland	Telephone and Email
RMD	Esbjerg, Denmark	Radio
C.I.R.M.	Rome, Italy	Telephone, Email, and Web applications

RMN: Radio Medico Norway; CCMM: Centre De Consultation Medicale Maritime; UCTM: University Center of Maritime and Tropical Medicine; RMD: Radio Medical Denmark; and C.I.R.M: Centro Internazionale Radio Medico.

**Table 3:** Study characteristics.

Study Type	Sample	Year	Medical Advice	Observations	Ref
Descriptive	551	2016	Management of cardiac symptoms	Pre-employment medical examinations improved preventive measures.	[21]
Observational	169	2017	Emergency helicopter evacuations (helivacs) between the two ferries	Every two weeks, one person was airlifted. The majority of Halifax was heart-related, with more cardiac cases airlifted than ambulances	[17]
Case study	5	2017	Diagnoses and follows up on medical conditions were carried out	In the development of telemedical technologies, the participants demonstrate a continual interest in teleconsultations with photographs	[23]
Observational	225	2019	Ensure permanent access to medical advice for seafarers	Providing medical assistance for various medical problems to seafarers requires close multidisciplinary cooperation between medical officers.	[18]
Retrospective cohort	1401	2019	Medical advice for injuries among seafarers	Danish-flagged merchant ships carry an increased risk of injuries to non-officers and European seafarers.	[22]
Retrospective	11,481	2020	Proposing prevention measures in COVID-19 Control	Fever, sore throats, and shortness of breath appeared to be more common during Coronavirus outbreaks	[8]
Epidemiological	423	2021	Assistance to control injuries and diseases among seafarers	Non-officers reported significantly more injuries and diseases than officers	[24]
Cross-sectional	420	2022	Diagnosis of marine workers’ dermatological diseases	Highlighted insufficient remote management of dermatological conditions.	[19]
Observational	384	2022	Diagnosis of COVID-19	Promotes social distancing and quarantine procedures at sea to limit the spread of the pandemic	[20]

## Data Availability

No new data were created or analyzed in this study. Data sharing is not applicable to this article.
